# Clinical study of treatment switching from premixed insulin to basal insulin combined with oral hypoglycemic drugs in patients with type 2 diabetes

**DOI:** 10.1186/1758-5996-6-37

**Published:** 2014-03-13

**Authors:** Ying Zhang, Yi-juan Xie, Dong-dong Meng, Hao-hang Zhang, Hui Chen, En Liu

**Affiliations:** 1 Department of Endocrinology, The Third Affiliated Hospital of Guangzhou Medical University, Guangzhou, Guangdong 510150, PR China

**Keywords:** Type 2 diabetes mellitus, Glargine, Premixed insulin, Oxidative stress

## Abstract

**Aim:**

Premixed insulin regimens are commonly used for the treatment of patients with type-2 diabetes mellitus (T2DM). However, limited data are available regarding next-step therapy options in cases where premixed insulin fails to provide adequate glycemic control. This 20-week observational study of everyday clinical practice evaluated the efficacy, safety and treatment satisfaction of insulin glargine plus oral anti-diabetic drugs (OADs) in T2DM patients previously treated with premixed insulin.

**Methods:**

In this open-label, single-arm, 20-week study, 70 subjects with T2DM inadequately controlled with premixed insulin were switched to insulin glargine plus OADs. Changes in glycaemic control, incidence of hypoglycaemia, treatment satisfaction using the Diabetes Treatment Satisfaction Questionnaire (DTSQ), serum superoxide dismutase (SOD), and serum 8-iso-prostaglandin (8-iso-PG) were evaluated at the start and the end of the study.

**Results:**

Over the 20 week treatment period, mean (±SD) HbA1c levels decreased from 8.28 ± 1.24% to 6.83 ± 1.09%, mean (±SD) FBG levels decreased from 7.64 ± 1.36 mmol/L to 5.57 ± 1.21 mmol/L, and 2 h PBG levels decreased from 12.07 ± 1.17 mmol/L to 8.94 ± 1.56 mmol/L, all *P* < 0.001. A total of 3 symptomatic hypoglycemic episodes were reported. No significant reductions in body weight were observed. The mean daily dose of insulin decreased by 14 U between week 0 (30.20 ± 9.93 U) and week 20 (16.38 ± 5.15 U). The total treatment satisfaction score showed a significant increase from study baseline to end point. Significant increases in SOD(90.00 ± 16.62 to 108.81 ± 27.02 u/ml, *P* < 0.01) and reductions in 8-iso-PG(2.15 ± 0.61 to 1.64 ± 0.42 pg/ml, *P* < 0.05) were observed between the start and end of the observation period. There were significant differences in baseline HbA1c, duration of diabetes, and baseline postprandial C-peptide between the A1c ≤ 6.5% group and the A1c > 7.0% group [HbA1c: 7.25% ± 1.02% *vs.* 9.32% ± 1.23%; duration: 7.84 ± 1.02 *vs.* 13.96 ± 1.35 years; postprandial C-peptide: 4.83 ± 2.11 *vs* 2.54 ± 0.87 nmol/L, all *P* < 0.05].

**Conclusions:**

The observational study shows that, in T2DM patients inadequately controlled with premixed insulin, switching therapy to glargine plus OADs is associated with significant improvements in glycaemic control and treatment satisfaction, and is with low incidence of hypoglycemia. Baseline postprandial C-peptide, HbA1c, and duration of diabetes are the key factors closely related to efficacy of this treatment regimen.

## Introduction

Type-2 diabetes mellitus (T2DM) is a progressive condition in which the level of glycated hemoglobin rises inexorably over time, and the function of beta cells declines. If lifestyle interventions and oral anti-diabetes drugs (OADs) prove inadequate in controlling glycemic levels, insulin therapy becomes necessary.

Until recently, opinion on how, or when, to start insulin treatment in T2DM was divided, and initial insulin treatment regimens are known to vary between different countries. Professional diabetes organizations recommend that basal or premixed insulin could be used as the initial insulin therapy. The basal insulin analogue glargine has a long duration of action (approximately 24 h), with little or no discernible peak in blood insulin concentration, and a lower rate of hypoglycemia. Combination therapy of OHA with basal insulin can be regarded as an effective first choice method for introducing insulin in a stepwise approach, adapting to the progressive β-cell failure [1]. Indeed, in China, many patients use premixed insulin as the initial insulin therapy, in part because premixed insulin has come to the market earlier, and is considerably cheaper than basal insulin analogs. Premixed insulin usually contains a rapid-acting insulin combined with an intermediate-acting insulin to mimic endogenous insulin secretion patterns, and is taken once or twice daily, normally before breakfast and dinner. The use of premixed insulin therapy could therefore result in a clinically relevant improvement in a patient’s glycated hemoglobin level. However, premixed insulin alone can be insufficient to achieve and sustain optimal glycemic control, and there are often additional concerns regarding hypoglycemia, weight gain, and lifestyle restrictions [2]. Moreover, there is limited information available regarding therapeutic options for patients for who premixed insulin provides inadequate glycemic control, who frequently experience episodes of hypoglycemia or those who want to avoid additional injections.

A currently favored hypothesis is that oxidative stress, through a single unifying mechanism of superoxide production, is the common pathogenic factor leading to insulin resistance, β-cell dysfunction, and T2DM [3]. Hyperglycemia and fluctuating blood glucose concentrations may also contribute to oxidative stress [4]. Therapies aimed at reducing oxidative stress may therefore benefit patients with T2DM.

A 12-week study in German [5] indicated that transferring patients with T2DM from premixed insulin to once-daily insulin glargine plus OADs provides a convenient, safe and effective treatment option that significantly improves metabolic control. However, whether these benefits extend to Chinese patients with Type 2 diabetes in a longer duration of time has yet to be investigated. In this study, we conducted a clinical trial to evaluate the efficacy and safety of insulin glargine with concomitant OADs in patients with T2DM who were previously sub-optimally controlled on premixed insulin therapies. The primary objective was to compare the change in A1c from baseline to endpoint (week 20). In addition, secondary objectives included the percentage of participants who achieved A1c of 6.5-7.0% or less, the number of hypoglycemic events, and the change in body weight, treatment satisfaction, and changes in biomarkers of oxidative stress. Furthermore, the study evaluated potential patient characteristics that may aid in identifying individuals who will benefit most from such a switch in regimens.

## Research design and methods

### Patients

The patients with a T2DM duration of at least one year were enrolled in the Endocrinology Department of the Third Affiliated Hospital of Guangzhou Medical University between Nov 2010 to Mar 2012. These patients, aged 18–75 years, were being treated with a stable dose of human or analogue premixed insulin (the most frequently prescribed ratios were 25/75 and 30/70), without oral hypoglycemic drugs, for at least 3 months. Further inclusion criteria included HbA1c levels between 7.5 and 10.0%. The decision to use a therapy regimen including insulin glargine with OADs was at the discretion of the physicians and patients, and depended partly on subjective parameters, including lack of efficacy of premixed insulin, the patient wanting a more flexible lifestyle, frequent occurrence of hypoglycemia with previous therapy, and lack of tolerance of the previous therapy. Exclusion criteria were according to the indications and contra-indications (such as patients hypersensitive to insulin glargine or any of the excipients) given in the prescribing information and summary of product characteristics for insulin glargine, patients treated with steroid or nonsteroidal anti-inflammatory drugs, patients who had experienced an acute concurrent illness during the 3-month period preceding the investigation, acute diabetic complications (such as diabetic ketoacidosis or hyperosmolar non-ketotic coma), severe chronic diabetic complications, and severe intercurrent illness. Additional exclusion criteria were sight-threatening retinopathy, a plasma creatinine level of 1.47 mg/dl (130 μmol/L) or more, cardiac disease (a history of unstable angina or myocardial infarction within the previous 6 months, or New York Heart Association class III or IV congestive heart failure), uncontrolled hypertension (systolic pressure ≥180 mmHg or diastolic pressure ≥105 mmHg), hepatic disease, an alanine aminotransferase level at least twice the upper limit of the normal range, tumors, and the likelihood of pregnancy.

The study was conducted in accordance with the declaration of Helsinki and Good Clinical Practice guidelines. Before study entry, all participants were willing to perform glucose self-monitoring, and all patients gave written informed consent.

### Study design

This 20-week trial was an open-label, non-interventional, observational study. At the start of the observation period, patients were given insulin glargine (Lantus, Sanofi–Aventis, Shanghai, China) to be administered once daily via subcutaneous injection; dosing decisions and concomitant OAD therapy (dosage, type and changes where necessary) were at the discretion of the physician. After the initial visit (week 0, start of observation), subsequent visits were scheduled at weeks 2, 4, 8, 12, 16 and 20 (end of observation), with interim telephone contact. Self-monitored blood glucose (SMBG) was performed using the patients’ own BG meter (Optium Xceed, Abbott). Physicians gave all patients training to ensure they could perform SMBG correctly and accurately. Before each visit and telephone contact, patients were asked to perform three capillary glucose profiles obtained before meals, 2 hours after eating, and at bedtime.

The trial-management system estimated initial doses of glargine according to the following formulae: for patients with an A1c < 8%, the initial doses of glargine was the total daily dose of premixed insulin × 0.6; for patients with an A1c ≥ 8%, the starting glargine dose was equal to total daily dose of premixed insulin × 0.8. During the first 4 weeks of the study, glargine doses were titrated every 3-4 days to reach the glycemic goal. This goal was defined as a fasting capillary blood glucose of less than 5.5 mmol/L, and capillary blood glucose at 2 h after each of three meals of less than 8.0 mmol/L. Glargine doses were adjusted by a forced titration regimen [6] (presented in Table 1).

**Table 1:** Insulin glargine dose titration algorithm and monitoring

Insulin dose titration algorithm	If self-monitored fasting BG for 2 consecutive days with no severe hypoglycemia:	
	> 8.9 mmol/l (> 160 mg/dl)	Add 8 IU/day
	>7.8 to ≤ 8.9 mmol/L(>140 to ≤160 mg/dl)	Add 6 IU/day
	>6.7 to ≤ 7.8 mmol/L(>120 to ≤140 mg/dl)	Add 4 IU/day
	>5.5 to ≤ 6.7 mmol/L(>100 to ≤120 mg/dl)	Add 2 IU/day
	5.5 mmol/l (≤100 mg/dl)	No further

During the observation period, blood pressure, heart rate, waist circumference, and body weight were measured, while body-mass index (BMI) was calculated at all visits. HbA1c, fasting C-peptide, 2 hour postprandial C-peptide, serum superoxide dismutase (SOD), and serum 8-iso-prostaglandin (8-iso-PG) were measured at the start and at the end of the observational period. HbA1c was measured by high-performance liquid chromatography (Biorad Variant II, Biorad), while serum C-peptide was assessed by chemilluminescence assay (Immulite, DPC, LA, USA). Serum SOD and 8-iso-PG were measured using enzyme-linked immunosorbent assay (Dako).

Treatment satisfaction was assessed using a diabetes treatment satisfaction questionnaire(DTSQ)with demonstrated validity and reliability in diabetic patient populations. The questions covered current satisfaction with the treatment, convenience of therapy, treatment flexibility, diabetes management (including weight), whether or not this treatment should be recommended to other patients, and whether to continue with the treatment regimen. Each factor is scored from 6 to 0 with a higher score indicating greater satisfaction; the treatment satisfaction score is the sum of the six items of the DTSQ for each respondent. Two additional factors measuring the perceived frequency of hypo- and hyperglycemia are scored from 0 (none of the time) to 6 (most of the time). DTSQ was administered at baseline and at end point.

Patients reported adverse events (AEs), including episodes of hypoglycemia, and adverse drug reactions (ADRs), either at each visit or at the time of occurrence. Hypoglycemia was defined as a blood glucose concentration <3.3 mol/L, with or without symptoms consistent with hypoglycemia. Hypoglycaemic episodes were defined as having classical symptoms of hypoglycaemia or blood glucose level below 3.3 mmol/L and prompt recovery after the patient self-administered carbohydrate. Severe hypoglycemia was defined either as an event with symptoms consistent with hypoglycemia, which required assistance, and was associated with a BG concentration of ≦2.0 mmol/l, or recovery after oral carbohydrate, intravenous glucose, or glucagon administration. Nocturnal hypoglycemia was defined as the occurrence of hypoglycemia while the individual was asleep. Whenever a participant awoke during the night and experienced symptoms of hypoglycemia, self-monitoring of BG was performed and documented in the patients’ diary.

### Statistical analysis

Data were analyzed using the SPSS 11.0 program, and statistical significance was determined using paired *t* test. To analyze the data that are not normally distributed, logarithmic transformation will be performed. Count data were analyzed with chi-square test. Significance was defined as *P* < 0.05.

## Results

### Patients

Of 78 patients who underwent screening, a total of 33 male and 37 female patients were enrolled in to the observation and completed the study. Age (mean ± SD), BMI, and duration of diabetes were 59.95 ± 10.21 years, 24.10 ± 3.87 kg/m^2^, and 7.78 ± 2.95 years, respectively. Duration of premixed insulin therapy was 3.21 ± 0.87 years. The breakdown of prior insulin therapies of the patient population is as follows: 61.43% Humalog Mix 75/25™ (Lilly, USA), 15.71% Humulin70/30 (Lilly, USA), 18.57% Novomix30 (Novo Nordisk, Denmark), and 4.29% Novolin30R (Novo Nordisk, Denmark). Among the patients, 8 patients treated with premix insulin from diagnosis of DM, 43 patients initiated insulin after failure of one or two OADs, 19 patients initiated insulin after failure of two or three OADs . The most common reasons given by physicians and patients for switching from premixed insulin to insulin glargine included lack of efficacy of premixed insulin (48.57%), the patient wanting a more flexible lifestyle (50.0%), frequent occurrence of hypoglycemia with the previous therapy (17.14%), and lack of toleration of the previous therapy (14.29%).

### Efficacy

**Figure 1:**
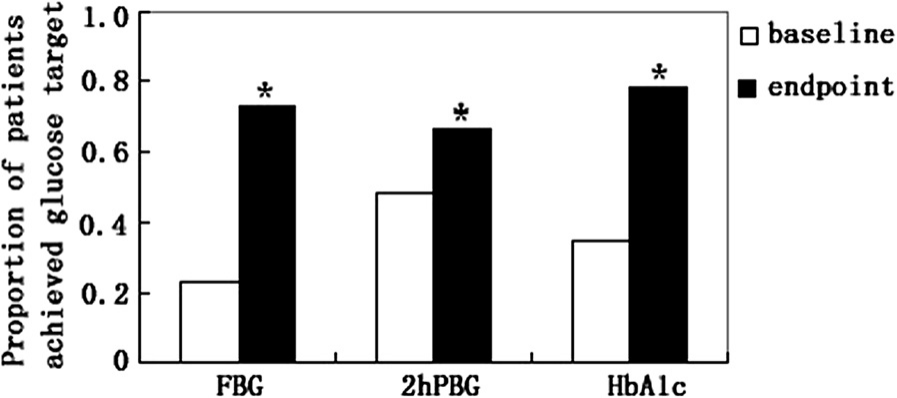
**Proportion of patients who achieved clinically relevant changes in FBG (4.1–5.5 mmol/l), 2 h PBG (7.2–8.9 mmol/l), or HbA1c (< 7.0%) target at week 20.** * *P* < 0.001 *vs.* baseline.

Over the 20-week treatment period, reductions in both BG(FBG, 2 h PBG)and HbA1c levels were observed. Mean (±SD) HbA1c levels decreased from 8.28 ± 1.24% to 6.83 ± 1.09%, mean (±SD) FBG levels decreased from 7.64 ± 1.36 mmol/L to 5.57 ± 1.21 mmol/L, and mean 2 hour PBG levels decreased from 12.07 ± 1.17 mmol/L to 8.94 ± 1.56 mmol/L. In total, 52 (74.29%) patients reached their target FBG levels of 4.1–5.5 mmol/l, while 44 (62.86%) patients achieved the target 2 h PBG level of 7.2–8.9 mmol/l. Compared with baseline, 56 (80.0%) patients obtained hemoglobin A1c concentration of 7% or less (Figure 1).

### Safety

During the 20-week treatment period, a total of 3 symptomatic hypoglycemic episodes were reported in 2 patients at 2 hours after breakfast. The lowest blood glucose level was 3.2 mmol/L. There were no nocturnal or severe hypoglycemia events reported.

### Body weight

A non-significant reduction in body weight was observed between the baseline and end of the observation period. Mean (±SD) body weight decreased from 61.19 ± 9.29 kg to 60.07 ± 9.16 kg (*P* >0.05), and BMI was stable at both baseline and the final visit.

### Insulin dose and oral anti-diabetic therapy

The mean daily dose of insulin decreased by 14 U between week 0 (30.20 ± 9.93 U) and week 20 (16.38 ± 5.15 U). At baseline, all patients were treated with premixed alone, while at the endpoint all patients were treated with insulin glargine and OADs. Among the OADs used, most patients (82.86% [n = 58]) received a single drug, while the rest (17.14% [n = 12]) received 2 oral combination therapies. Metformin, glimepiride, glibenclamide, and α-glucosidase inhibitors were used most frequently.

### Patient satisfaction

The total treatment satisfaction score (DTSQ score) showed a significant increase from baseline to end point during treatment with glargine + OAD, increasing from 27.12 ± 4.29 to 32.07 ± 5.16 (*P* <0.05). Patients were found to significantly favor the switched treatment for five of six items in the treatment satisfaction scale: current satisfaction with treatment (3.4 ± 0.8 *vs.* 5.1 ± 0.5; *P* <0.01), convenience of treatment (3.1 ± 0.5 *vs.* 4.9 ± 0.2; *P* < 0.01), flexibility of treatment (2.9 ± 0.5 *vs.* 5.2 ± 0.4; *P* <0.01), recommend to others (3.3 ± 0.8 *vs.* 5.0 ± 0.2; *P* < 0.01), and satisfaction to continue current treatment (3.4 ± 0.9 *vs.* 5.2 ± 0.4; *P* <0.01). No significantly changes in scores of diabetes management (including weight) were recorded (4.7 ± 0.4 *vs.* 4.5 ± 0.6, *P* > 0.05). Moreover, the DTSQ showed a decrease in perceived frequency of hypoglycemia (3.4 ± 0.2 *vs.* 0.8 ± 0.3, *P* < 0.01) and hyperglycemia (3.2 ± 0.7 *vs*. 1.5 ± 0.3, *P* < 0.01) during the observation period.

### Identification of factors associated with reaching target HbA1c values

Differences between the patient groups who did and did not reach HbA1c targets (A1C ≤ 6.5%, 6.5% < A1C <7% or A1C >7.0%) by study end were compared. The age, BMI,weight,WHR,fasting C-peptide at baseline, and premixed insulin dose were similar between the three groups. The duration of diabetes was significantly longer in A1C > 7.0% group than in the A1C ≤ 6.5% group (*P* < 0.01), and the level of baseline HbA1c, baseline 2-hour postprandial C-peptide were significantly higher in A1C > 7.0% group than in the A1C ≤ 6.5% group (*P* < 0.01). The A1C ≤ 6.5% group had shorter duration of diabetes, higher baseline 2-hour postprandial C-peptide and baseline HbA1c. After 20 weeks treatment, the poorly controlled group (A1C > 7.0% at end point) had a more marked reduction in HbA1c. The two OADs combination therapies was more frequently used in the A1C > 7.0% group (A1C ≤ 6.5% group n = 0; A1C > 7.0% group n = 10; *P* = 0.000) Table 2.

**Table 2:** Baseline characteristics, clinical presentation, with different controlled glycemia

**Characteristic**	**≤6.5%**	**Between 6.5% and 7%**	**>7%**	***P***
Number	16	39	15	
age(yr)	58.31 ± 7.21	59.30 ± 6.87	57.33 ± 8.23	0.652
Duration of diabetes(yr)	7.84 ± 1.02	8.01 ± 0.97	13.96 ± 1.35	0.012^#^
Baseline BMI(kg/m^2^)	24.11 ± 3.34	24.87 ± 3.56	23.98 ± 4.56	0.143
Baseline weight (kg)	61.35 ± 8.76	60.04 ± 9.13	58.23 ± 3.58	0.087
Baseline WHR	0.89 ± 0.09	0.90 ± 0.12	0.86 ± 0.15	0.321
Baseline HbA1c(%)	7.25 ± 1.02	8.11 ± 0.85	9.32 ± 1.23	0.028^#^
Baseline fasting C-peptide	1.82 ± 0.65	1.87 ± 0.78	1.84 ± 0.53	0.083
Baseline 2-h postprandial C-peptide	4.83 ± 2.11	3.89 ± 1.15	2.54 ± 0.87	0.034^#^
Baseline premixed insulin dose(U/d)	30.50 ± 4.12	30.23 ± 7.65	33.87 ± 8.21	0.056
HbA1c change from baseline	0.82 ± 0.10	1.42 ± 0.33	1.62 ± 0.58	0.008*
Concomitant OADs during the study n (%)				
Metformin	4(5.71)	8(11.43)	0(0.00)	
SUs	7(10.00)	18(25.71)	3(4.29)	
α-glucosidase	5(7.14)	11(15.71)	2(2.86)	
Met/SUs	0(0.00)	2(2.86)	6(8.57)	
Met/α-glucosidase	0(0.00)	0(0.00)	2(2.86)	
SUs/α-glucosidase	0(0.00)	0(0.00)	2(2.86)	

*P < 0.01 ; ^#^*P* < 0.05, HbA1c > 7% group *vs.* HbA1c ≤ 6.5% group.

### Changes in markers of oxidative stress

Over the 20-week treatment period, significant increases in serum SOD, and significant reductions in serum 8-iso-PG were observed (Table 3).

**Table 3:** Comparison of serum SOD and 8-iso-PG between baseline and endpoint

	**Baseline**	**Endpoint**	***P***
SOD(u/ml)	90.00 ± 16.62	108.81 ± 27.02	0.009
8-iso-PG (pg/ml)	2.15 ± 0.61	1.641 ± 0.42	0.012

## Discussion

Here, we describe a non-interventional, non-randomized observational study that was undertaken to document the experience of transferring patients with T2DM from premixed insulin, to insulin glargine.

Several barriers exist for the initiation and subsequent optimization of insulin therapy, including the risk of hypoglycemia, weight gain, and concerns about daily injections and/or restrictions to lifestyle [7-9]. Ideally, an insulin regimen should be matched to the individual patient, considering his or her lifestyle needs and physical and mental capabilities. At the point of initiation of insulin treatment alone, the guidance and information provided varies significantly. AACE recommendations state that any one of the four different approaches to insulin initiation may be used, including a once-daily basal insulin regimen, a premixed insulin preparation that can be administered once or twice daily, a basal-bolus regimen, or a prandial regimen [10]. The published joint position statements from the ADA and EASD, however, only recommend the use of basal insulin in the first instance [11]. However in China, the use of premixed insulin as the initial insulin treatment is a popular option. We therefore set out to determine it is possible to improve treatment efficacy in the patients who were poorly controlled with premixed insulin, using basal insulin glargine + OADs.

Our study demonstrated that initiation of insulin glargine with OADs improves glycemic control in patients with T2DM who were poorly controlled with premixed insulin prior to the observation period. Patients were enrolled for varying reasons including lack of efficacy of premixed insulin (7% < HbA1c < 10%), wanting a more flexible lifestyle, frequent occurrence of hypoglycemia with previous therapy, and lack of tolerability of the previous therapy. During the 20 weeks of observation, significant improvements in both HbA1c and BG were observed. Furthermore, the daily insulin dose was decreased with no significant variation in body weight. Three episodes of symptomatic hypoglycemia occurred, but there were no episodes of severe hypoglycemia.

The premixed insulin products are composed of various fixed-ratio mixtures, with a percentage of rapid-acting insulin available for prandial phases, and the remainder of the mixture consisting of a protamine suspension of the insulin analog for basal periods. Randomized trials directly comparing the initiation of insulin therapy between basal and premixed analogues are scarce. The 4 T study compared three different insulin regimens: basal insulin once daily, premixed insulin twice daily or prandial insulin thrice daily. In this study the HbA1c at the end of the first year was similarly, the rates of hypoglycemia was lowest in the basal insulin group [12]. During the subsequent 2-year study extension, there was no statistical difference in the mean HbA1c among the three groups. Based on lower overall rates of minor hypoglycemia, reduced weight gain, and perceived convenience, the authors of this study recommended once-daily basal insulin as the first-line insulin therapy [13]. Janka et al. demonstrated that basal insulin glargine once daily, with glimepiride plus metformin treatment, was safer and more effective than beginning twice-daily injections of 70/30 and discontinuing OADs in type 2 diabetic patients inadequately controlled with OADs [14]. In a 12 week observational study of everyday clinical practice, Harmer et al. have shown that once daily insulin glargine plus OADs is an effective therapeutic regimen with a good safety profile for patients with T2DM who were inadequately controlled with premixed insulin [5]. A recent systematic review and meta-analysis of basal and premixed regimens demonstrated that twice-daily premixed insulin regimens reduced HbA1c by an additional 0.45% (CI 0.19–0.70%), but with additional weight gain and higher rates of hypoglycemia [15].

Glargine is a long-acting basal insulin analogue that has proved superior to NPH insulin for the management of T2DM. It can achieve a peakless level for at least 24 hours, so is associated with a lower risk of hypoglycaemia. This is of particular interest because fears of hypoglycemia remains one of the key obstacles to both initiating and optimizing insulin therapy. Premixed insulins are pre-made combinations of NPH and short-acting insulins. The action profile of NPH features a slightly higher peak than those of long-acting analogs. This increases the risk for hypoglycemia, especially interprandial and nocturnal hypoglycaemia, thus hindering the achievement of good metabolic control [16-18].

Before the switching treatment, all patients were treated with premixed insulin alone, and at the end of the study, all patients were treated with insulin glargine and OADs. It should be stressed that the amount of premixed insulin was not large ( approx. 30 IU/day) and patients have not been treated with OAD before switching. After switching, the use of OADs may contribute to achieving adequate glycemic control through different mechanisms.

Slight reductions in body weight were observed between the start and end of the observation period. Insulin is a growth-promoting hormone that promotes the storage of essential nutrients, including fats. A potential side effect of weight gain from insulin treatment can be troublesome, and can also promote insulin resistance, result in poorly controlled diabetes. In this study, the mean daily dose of insulin decreased by 14 U between week 0 and week 20, which may have contributed to the weight loss and well-controlled BG.

The basal insulin regimen was associated with a greater improvement in quality of life and treatment satisfaction compared with the previous premixed insulin. Fewer insulin injections with glargine therapy may have contributed to the greater treatment satisfaction. Other factors, such as oral medication, may also be involved in improving the treatment satisfaction. Pharmacological therapy alone is not enough to guarantee amelioration of patient condition, and controlling diabetes requires frequent monitoring by the patient, and for this reason compliance is vital to achieving euglycemia. The choice of any regimen depends to a large degree on whether the patient can conform to the complexity of the regimen over the long term. If switching a therapy improves patient compliance**,** it will lead to more favorable outcomes in diabetes.

When the sub-group of patients switched to glargine treatment is analyzed specifically, baseline postprandial C-peptide, HbA1c and diabetes duration are the key factors that are closely related to treatment success. The UKPDS has shown that β-cell function progressively deteriorates over time in patients with T2DM, irrespective of lifestyle and existing pharmacological interventions. The 2-hour postprandial C-peptide level represents the insulin response after a glucose challenge, which was used to assess β-cell-stimulated insulin secretory capacity. Patients who had lower HbA1c levels, a shorter duration of DM, and higher postprandial C-peptide levels had more chance to achieve glycemic control after switching treatment. For patients with reduced β-cell stimulated insulin–secretory capacity, represented by postprandial C-peptide, the benefits of glargine were limited. Even if majority of patients in the A1c >7.0% group taking two OADs, blood sugar of the patients was unsatisfactorily controlled, further prandial intervention was therefore required in these patients.

At 20 weeks, FBG levels were not significantly different among the 3 subgroups. However, the morning postprandial glucose, postprandial glucose values at noon and at dinnertime were statistically higher in the HbA1c > 7.0% subgroup than in HbA1c < 6.5% subgroup. The decrease in HbA1c was more obvious in the HbA1c > 7.0% subgroup than in the HbA1c < 6.5% group. This suggests that for the patients with poor glycemic control, the therapy using insulin glargine focused on FBG has a greater impact on overall glycemic control. The present study confirms previous reports that PBG contributes more to glycemic control in patients with mild or moderate hyperglycemia than in those with poorly controlled diabetes mellitus, in whom fasting hyperglycemia is the main contributor to overall hyperglycemia. Fasting hyperglycemia is the main contributor to glycemic load if HbA1c is above 8.5% [19].

Diabetes is characterized by glycemic disorders that include both sustained chronic hyperglycemia and acute glucose fluctuations. Oxidative stress, through the production of reactive oxygen species (ROS), has been proposed as the root cause underlying the development of insulin resistance, β -cell dysfunction, and T2DM [3]. Increased glycemic variability leads to the generation of more ROS and, despite having similar HbA1c levels. At present, most of the interventional trials using the “treat-to-target” concept have been conducted to achieve near-normal glucose value at fasting and A1C levels < 7%. Acute glucose fluctuations have rarely been taken into account. It was demonstrated that urinary excretion rate of 8-iso-PGF2 and serum 8-iso-PG, serum SOD were correlated with the glycemic variability and oxidative stress [20]. To ascertain the changes in oxidative stress during the switching treatment, the oxidative stress biomarker serum 8-iso-PG and the antioxidative stress biomarker serum SOD were measured. Through the period of observation is too short, significant increases in SOD and reductions in 8-iso-PG were observed between the start and end of the observation period. This suggests that glargine with OADs is the ideal regimen, improving glycemic control while probably minimizing blood glucose fluctuations.

In summary, in this observational study we have shown that once daily insulin glargine plus OADs is an effective therapeutic regimen with a good safety profile for patients with T2DM who were inadequately controlled with premixed insulin. Baseline postprandial C-peptide, HbA1c and diabetes duration are the key factors that are closely related to efficacy of this switching regimen.

## Competing interests

The authors declare that they have no competing interest.

## Authors’ contributions

YZ conducted the research, performed the statistical analyses and wrote the manuscript; YX, DM and HZ participated in the data collection and checked the data; HC and EL participated in the analysis of data and in the writing of the manuscript. All authors have read and approved the final manuscript.
